# Exploratory analysis on the relationship between dietary live microbe intake and arthritis: a national population based cross-sectional study

**DOI:** 10.3389/fnut.2023.1328238

**Published:** 2024-01-04

**Authors:** Yiping Liu, Yida Xing, Xiaodan Kong

**Affiliations:** Department of Rheumatology, The Second Affiliated Hospital of Dalian Medical University, Dalian, China

**Keywords:** microbiological therapy, dietary live microbe, rheumatoid arthritis, osteoarthritis, cross-sectional study

## Abstract

**Objective:**

The pathogenesis of arthritis such as rheumatoid arthritis (RA) and osteoarthritis (OA) remains unclear. This study aims to investigate whether the intake of live dietary microbes can be used as an auxiliary means for the treatment of arthritis.

**Methods:**

Data used in the present research were originated from the US National Health and Nutrition Examination Survey (NHANES) from 2003–2018. Participants involved in the present study were categorized into three groups based on the dietary live microbe classification system, namely low, medium, and high dietary live microbe groups. The analyses utilized weighted univariate and multivariate logistic regression. The restricted cubic spline plot was used to explore the relationship between the high dietary live microbe group and the odds of arthritis.

**Results:**

12,844 participants were included in the present study. The intake of high live dietary microbes in RA group was lower than that in healthy control group and OA group. The proportion of RA patients in the high live dietary microbe group was lower than that in the low and medium live dietary microbe group. Following the comprehensive adjustment for covariates, it was observed that participants in both the high and medium dietary live microbe groups exhibited lower odds of RA compared to those in the low dietary live microbe group (High OR: 0.71, 95% CI: 0.53–0.96; Medium OR: 0.77, 95% CI: 0.59–1.00, *p* = 0.02). A restricted cubic spline plot indicates a negative correlation between the quantity of dietary live microbes and the occurrence of RA within the high dietary live microbe group.

**Conclusion:**

The results of our study revealed a significant difference in dietary live microbe intake between healthy and RA patients. Higher dietary intake was correlated with a decreased odds of RA. However, no significant association was found between the occurrence of OA and the quantity of dietary live microbes.

## 1 Introduction

As societal industrialization progresses and individuals experience enhanced living conditions, there is an increasing focus on environmental and food sanitation, resulting in a decline in the abundance and diversity of microorganisms. Undoubtedly, the improvement of sanitary conditions would bring obvious benefits to public health, however, it would also give rise to novel challenges, including the emergence of immune-related ailments resulting from diminished exposure to microorganisms. Some studies have demonstrated that the consumption of viable and non-harmful dietary microbes might have a positive impact on enhancing overall health (1). The “old friend” theory posits that innocuous or mutually beneficial bacteria encountered in everyday life have the potential to enhance immune system regulation through interactions with the intestinal mucosa, thus diminishing vulnerability to diseases (2). This has also been verified in mice that the local and systemic immune systems of mice raised under aseptic conditions are not fully developed (3). Therefore, as a crucial determinant in shaping the composition of intestinal microflora, the significance of dietary living microbes in chronic inflammatory diseases cannot be disregarded (4).

Rheumatoid arthritis (RA) is a common systemic autoimmune disease with a prevalence rate of about 0.5–1% (5). It is characterized by systemic inflammation, persistent synovitis, and progressive joint destruction, often accompanied by involvement of extra-articular organs, which also have the possibility of joint deformity and loss of function (6). Osteoarthritis (OA) is a degenerative disease in middle-aged and elderly people that is characterized by many pathological changes, including synovitis, reactive hyperplasia of the articular border and subchondral bone, degradation and loss of articular cartilage, and potential impairment of mobility (7, 8). The specific pathogenesis of these two diseases remains unclear; nevertheless, existing research indicates that factors such as nutrition, environment, and microbial agents may exert significant influence in their development (8). These factors can participate in the pathogenesis of RA by triggering the activation of specific human leukocyte antigen (HLA) DRB1 alleles in genetically susceptible individuals (9, 10). There exists a notion positing that the increase in intestinal permeability will lead to the leakage of intestinal microbes and the translocation of exposed intestinal microbes to the adjacent joints, ultimately contributing to the occurrence of RA (9). Probiotics may regulate immune balance by affecting intestinal flora balance, intestinal permeability, mucosal inflammation and cytokine release, thus reducing the risk of RA (9). Some studies have also shown that intestinal microbes and their related components and metabolites may be involved in the pathogenesis of OA by activating local or systemic innate immune responses. However, the precise mechanism behind this association remains unclear (11, 12). Compared with tumors, metabolic diseases and nervous system diseases, the distribution and changes of intestinal microbes in autoimmune diseases are more consistent, which is of great significance for the prediction of autoimmune diseases (13).

Given the evident adverse effects associated with existing clinical medications utilized in arthritis treatment (14), it is necessary to investigate whether the intake of live dietary microbes can be used as an auxiliary means for the treatment of arthritis. By comparing the incidence of arthritis among people with different dietary habits in daily life, this paper aims to illustrate the relationship between dietary live microbes and the odds of arthritis, and seeks to explore the possible adjuvant role of dietary live microbes in autoimmune diseases.

## 2 Methods

### 2.1 Study design and population

**Figure 1:**
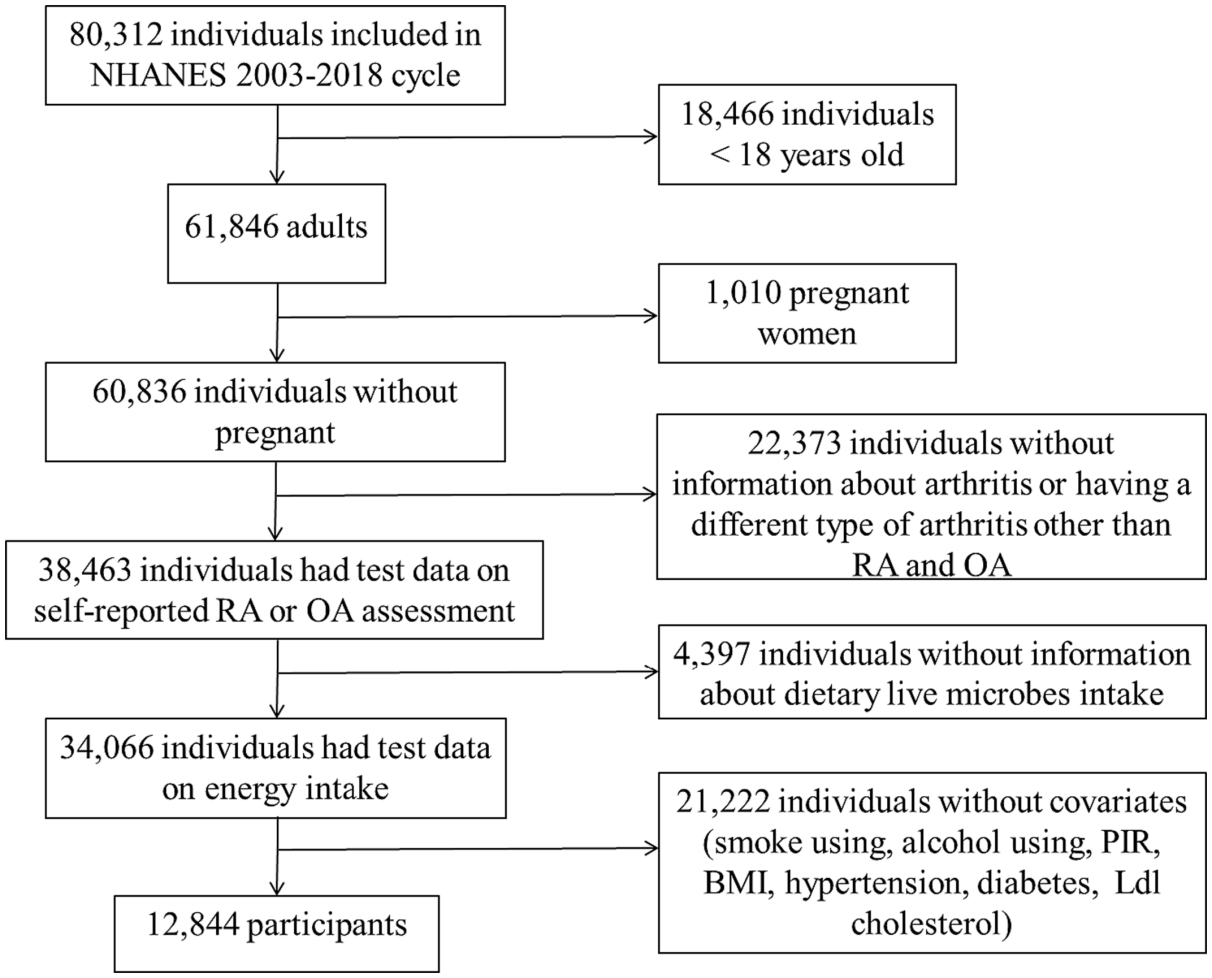
Flow chart of the study subjects, NHANES 2003–2018.

The National Health and Nutrition Inspection Survey (NHANES) is a survey conducted by the National Center for Health Statistics (NCHS) to assess the nutrition and health status of non-institutionalized American civilians. The NHANES plan was approved by the NCHS Institutional Review Board, and all participants in the survey signed informed consent (15). This study included a total of 80,312 participants, spanning eight consecutive cycles from 2003–2004 to 2017–2018. Among these participants, 18,466 were under 18 years old, 1,010 were pregnant, 22,373 lacked diagnostic information on arthritis (including uncertainty about having arthritis or having a different type of arthritis other than RA and OA), 4,397 lacked information on dietary intake of live microbes, and 21,222 had missing data on core covariates (Supplementary Table 1). In conclusion, a total of 12,844 participants were incorporated into the study (Figure 1).

### 2.2 Assessment of arthritis

The arthritis of the subjects was evaluated using a self-reported questionnaire. The evaluation methods employed in this study involve the use of specialized inquiry methods, wherein participants are presented with a set of three distinct questions: Has a doctor or other health professional ever told you that you had arthritis? How old were you when you were first told had arthritis? Which type of arthritis was it? The individuals diagnosed with RA and OA were included in the study based on the aforementioned three screening questions.

### 2.3 Dietary live microbe intake

The NHANES database collects dietary information from participants through in-person interviews, specifically focusing on the details of their food intake within a 24-h period. The NHANES cycle-specific Food and Nutrient Database for Dietary Studies of the USDA was used to determine the amount of energy and nutrients contained in each individual food item and beverage that was consumed (16, 17). According to the number of live microbes per gram of diet determined by experts on 48 subgroups and 9,388 foods and according to the content of microbes per gram of food, MLM, MES, RH, and CH (experts in the field) classify food into three categories based on variations in live microbes found in different foods. This classification is determined by referencing relevant literature, authoritative comments, or inferred based on the food processing methods used (18–21). This classification can reflect the approximate number of microbes expected to survive in different foods (22). Foods with different live microbes were divided into low (<10^4^ CFU/g), medium (10^4^–10^7^ CFU/g), and high (>10^7^ CFU/g): Level of low refers to food that has undergone the process of pasteurization. Level of medium denotes fresh fruits and vegetables that have not been peeled. Lastly, level of high represents unpasteurized fermented food and probiotic supplements (22).

### 2.4 Covariates

According to the previously published articles and relevant clinical experience (23), the confounding factors that may affect the relationship between dietary live microbes and arthritis were controlled. The main covariates are as follows: Age is categorized into two groups: < 60 years old, and 60 years old and above. Gender is classified as male or female. Race is categorized into four groups: Mexican American, non-Hispanic black, non-Hispanic white, and other. The individual’s marital status can be categorized into the following classifications: married or cohabiting, widowed, divorced, separated, or never married. The poverty-income ratio (PIR) is categorized into three groups: lower income, which refers to individuals or households with an income equal to or less than 1.35%; moderate income, which includes those with an income ranging from 1.35 to 3.5%; and higher income, which encompasses individuals or households with an income equal to or greater than 3.5%. The Body Mass Index (BMI) is classified into two categories: normal (BMI < 25.0 kg/m2), overweight/ obesity (BMI ≥ 25.0 kg/m2). Alcohol consumption is categorized as either ever/current (having consumed ≥12 drinks in one’s lifetime) or never (having consumed <12 drinks in one’s lifetime). Smoking status is divided into never smokers (smoked less than 100 cigarettes in life), former/ current smokers (smoked more than 100 cigarettes in life). Hypertension is diagnosed by self-reported history of hypertension, measurement of blood pressure (over 140/90 mm Hg per year), or use of antihypertensive medication. Diabetes is defined as any of the following: HbA1c level ≥ 6.5%, serum glucose at 2 h following a 75 g glucose load (OGTT) or random blood glucose level ≥ 11.1 mmol/L, fasting glucose level ≥ 7.0 mmol/L, self-reported diagnosis of diabetes, or self-reported use of insulin or other diabetes medication.

### 2.5 Statistical analysis

In order to better adjust the impact of unequal selection probabilities, oversampling of certain subgroups and non-response of selected samples on the real situation, and to ensure nationally representative estimates, we used survey-specific sample weights for the complex sampling design. The distribution features were established using the mean and standard deviation (SD) for continuous data, while the number of cases (n) and percentage (%) were employed for categorical variables. The chi-square test was employed to evaluate disparities in categorical data, while disparities in continuous variables were evaluated using either the t-test (for variables with a normal distribution) or the Mann–Whitney test (for variables with a skewed distribution). The participants in this study were categorized into three groups: The low dietary live microbe group consisted of individuals who consumed all foods with a low quantity of live microbes. The medium dietary live microbe group included individuals who consumed foods with a medium number of live microbes, excluding those with high live microbe content. The high dietary live microbe group comprised individuals who consumed any foods with a high quantity of live microbes. Weighted univariate and multivariate logistic regression methods were employed to assess the association between dietary live microbes and the odds of developing inflammatory arthritis. Three distinct models have been established for this purpose. In Model 1, the adjustment was made solely for age and gender variables. In Model 2, further adjustments were made for Race/ethnicity, marital status, PIR, and BMI. Model 3 included additional adjustments for variables such as diabetes, hypertension, smoking status, alcohol usage, Ldl cholesterol, and energy intake. Restricted cubic spline regression analysis was used to explore the relationship between the high dietary live microbe group and the odds of RA. Additionally, subgroup analyses were also conducted by age, sex, BMI, smoking status, alcohol use, hypertension, and diabetes. All statistical analysis is carried out with R software (version 4.3.1).

## 3 Results

### 3.1 Baseline information on people who consume different doses of dietary live microbes

12,844 participants were included in this study, including 4,549 in the low dietary live microbe group, 5,386 in the medium dietary live microbe group, and 2,909 in the high dietary live microbe group. Table 1 shows the weighted baseline characteristics of participants in the low, medium, and high dietary live microbe groups in the NHANES database from 2003 to 2018. Significant differences among low, medium, and high dietary live microbe groups were observed in all parameters except Ldl cholesterol. Among the studied population, participants under the age of 60, females, and individuals with a high PIR had a higher consumption of dietary live microbes. Compared with the low and medium dietary live microbe groups, the high dietary live microbe group contained fewer people with overweight, obesity, diabetes, and hypertension. It is noteworthy that the group with high dietary live microbes had the lowest odds of RA patients and the highest odds of OA patients.

**Table 1:** The clinical characteristics of the study population in relation to various dietary live microbes.

	Low Dietary Live Microbe Group (*n* = 4,549)	Medium Dietary Live Microbe Group (*n* = 5,386)	High Dietary Live Microbe Group (*n* = 2,909)	*p*-value
**Age (%)**				**< 0.001**
<60	3,255 (79.48)	3,512 (71.48)	2055 (74.79)	
> = 60	1,294 (20.52)	1874 (28.52)	854 (25.21)	
**Gender (%)**				**< 0.001**
Female	2077 (44.61)	2,695 (51.64)	1,576 (54.29)	
Male	2,472 (55.39)	2,691 (48.36)	1,333 (45.71)	
**Race/ethnicity (%)**				**< 0.001**
Mexican American	640 (7.24)	1,063 (10.10)	383 (6.06)	
Non-Hispanic Black	1,251 (15.79)	947 (9.26)	361 (5.63)	
Non-Hispanic White	1830 (64.53)	2,418 (68.35)	1,619 (78.16)	
Other	828 (12.44)	958 (12.29)	546 (10.15)	
**Marital status (%)**				**< 0.001**
Married/cohabiting	2,514 (56.01)	3,408 (65.06)	1848 (66.83)	
Widowed/divorced /separated/ never married	2025 (43.99)	1978 (34.94)	1,061 (33.17)	
**PIR (%)**				**< 0.001**
≤1.35%	1,685 (29.29)	1,567 (19.77)	675 (15.69)	
1.35–3.5%	1769 (37.22)	2023 (35.48)	997 (30.90)	
≥3.5%	1,095 (33.49)	1796 (44.75)	1,237 (53.41)	
**BMI (%)**				**0.005**
Normal	1,342 (30.00)	1,611 (32.29)	960 (34.97)	
Overweight/ Obesity	3,207 (70.00)	3,775 (67.71)	1949 (65.03)	
**Alcohol using (%)**				**0.004**
Ever/Current	3,934 (88.92)	4,655 (88.58)	2,578 (91.61)	
Never	615 (11.08)	731 (11.42)	331 (8.39)	
**Smoking status (%)**				**< 0.001**
Now/ Former	2,203 (50.08)	2,344 (44.12)	1,208 (42.47)	
Never	2,346 (49.92)	3,042 (55.88)	1701 (57.53)	
**Diabetes (%)**				**< 0.001**
No	3,725 (85.81)	4,305 (83.95)	2,464 (88.67)	
Yes	824 (14.19)	1,081 (16.05)	445 (11.33)	
**Hypertension (%)**				**0.02**
No	2,669 (62.42)	3,177 (62.23)	1846 (66.22)	
Yes	1880 (37.58)	2,209 (37.77)	1,063 (33.78)	
**Energy take(kcal)**	2092.18 ± 18.42	2165.82 ± 19.57	2288.40 ± 23.81	**< 0.001**
**Ldl cholesterol (mg/dl)**	114.00 ± 0.76	114.75 ± 0.68	115.02 ± 1.01	0.71
**Arthritis type (%)**				**< 0.001**
RA	318 (6.00)	327 (4.68)	141 (3.48)	
OA	479 (11.52)	693 (13.68)	405 (14.74)	
HC	3,752 (82.48)	4,366 (81.65)	2,363 (81.79)	

### 3.2 Baseline characteristics of the included population

The information about healthy individuals as well as patients diagnosed with RA and OA is shown in Table 2. Except for Ldl cholesterol, notable variations were observed in all indexes across the three groups. Compared to the healthy subjects, the groups diagnosed with RA and OA exhibited a greater proportion of female participants and those over 60 years old, a higher prevalence of overweight or obesity, a higher incidence of diabetes, and a higher prevalence of hypertension. In contrast to healthy individuals, patients diagnosed with RA exhibited a lower intake of high dietary live microbes, whereas patients with OA demonstrated a higher consumption of high dietary live microbes.

**Table 2:** The clinical characteristics of the study population of arthritis and health control.

	HC (*n* = 10,481)	RA (*n* = 786)	OA (*n* = 1,577)	*p*-value
**Age (%)**				**< 0.001**
<60	7,967 (81.92)	326 (51.30)	529 (40.41)	
> = 60	2,514 (18.08)	460 (48.70)	1,048 (59.59)	
**Gender (%)**				**< 0.001**
Female	4,899 (47.67)	436 (54.67)	1,013 (63.79)	
Male	5,582 (52.33)	350 (45.33)	564 (36.21)	
**Race/ethnicity (%)**				**< 0.001**
Mexican American	1842 (8.99)	114 (6.30)	130 (2.97)	
Non-Hispanic Black	2,106 (10.64)	228 (17.06)	225 (5.97)	
Non-Hispanic White	4,494 (67.78)	345 (67.52)	1,028 (83.66)	
Other	2039 (12.60)	99 (9.13)	194 (7.40)	
**Marital status (%)**				0.05
Married/cohabiting	6,368 (61.98)	456 (65.35)	956 (65.98)	
Widowed/divorced /separated/never married	4,113 (38.02)	330 (34.65)	621 (34.02)	
**PIR (%)**				**< 0.001**
≤1.35%	3,207 (21.69)	306 (28.96)	414 (18.87)	
1.35–3.5%	3,888 (34.39)	288 (39.78)	613 (35.23)	
≥3.5%	3,386 (43.92)	192 (31.26)	550 (45.89)	
**BMI (%)**				**< 0.001**
Normal	3,411 (34.39)	171 (25.53)	331 (21.90)	
Overweight/obesity	7,070 (65.61)	615 (74.47)	1,246 (78.10)	
**Alcohol using (%)**				0.05
Ever/Current	9,140 (89.84)	658 (85.83)	1,369 (88.96)	
Never	1,341 (10.16)	128 (14.17)	208 (11.04)	
**Smoking status (%)**				**< 0.001**
Now/former	4,471 (43.20)	453 (64.64)	831 (53.26)	
Never	6,010 (56.80)	333 (35.36)	746 (46.74)	
**Diabetes (%)**				**< 0.001**
No	8,848 (88.21)	522 (72.59)	1,124 (76.12)	
Yes	1,633 (11.79)	264 (27.41)	453 (23.88)	
**Hypertension (%)**				**< 0.001**
No	6,888 (68.91)	269 (39.12)	535 (38.18)	
Yes	3,593 (31.09)	517 (60.88)	1,042 (61.82)	
**Energy take(kcal)**	2223.78 ± 13.98	1936.07 ± 40.89	1972.02 ± 27.10	**< 0.001**
**Ldl cholesterol (mg/dl)**	114.77 ± 0.53	113.89 ± 1.94	113.69 ± 1.18	0.66
**Dietary live microbe group (%)**				**< 0.001**
Low	3,752 (32.02)	318 (40.08)	479 (27.58)	
Medium	4,366 (40.10)	327 (39.52)	693 (41.43)	
High	2,363 (27.88)	141 (20.39)	405 (30.98)	

### 3.3 Relationship between dietary viable microbe content and RA

The results of both univariate and multivariate weighted logistic regression analysis are presented in Table 3. The results from the univariate logistic regression analysis indicated that the odds of RA was significantly lower in the group with a high dietary intake of live microbes compared to the group with a low dietary intake of live microbes [odds ratio (OR) = 0.58, 95% confidence interval (CI) = (0.44, 0.78)]. On this basis, multivariate logistic regression analysis was carried out by controlling the relevant variables. In model 1, adjustments for age and gender were made, and compared to the group with low dietary live microbes, both the medium and high dietary live microbe groups exhibited decrease odds. Moreover, the high dietary live microbe group showed a more significant decrease in the odds of RA [Medium: OR 0.67, 95% CI (0.52, 0.87), High: OR 0.52, 95% CI (0.39, 0.70)]. In model 2, after further adjustment of race/ethnicity, marital status, PIR, and BMI, the odds remained lower in the medium and high dietary live microbe groups compared to the low dietary live microbe group [Medium: OR 0.75, 95% CI (0.58, 0.98), High: OR 0.63, 95% CI (0.47, 0.85)]. Model 3 further adjusts diabetes, hypertension, smoking status, alcohol usage, Ldl-cholesterol, energy take. It was observed that there was a significant statistical difference in the reduction of the odds between the groups with medium and high dietary live microbes as compared to the group with low dietary live microbes [Medium: OR 0.77, 95% CI (0.59, 1.00), High: OR 0.71 95% CI (0.53, 0.96)]. In both the crude model and the model adjusted for related variables, a statistically significant inverse association was observed between the consumption of live microbes in the diet and the odds of RA.

**Table 3:** Association between levels of Dietary Live Microbe and RA.

	Crude model	Model 1	Model 2	Model 3
Low Dietary Live Microbe Group OR (95% CI)	1.00	1.00	1.00	1.00
Medium Dietary Live Microbe Group OR (95% CI)	0.79 (0.61,1.01)	**0.67 (0.52,0.87)**	**0.75 (0.58,0.98)**	**0.77 (0.59,1.00)**
High Dietary Live Microbe Group OR (95% CI)	**0.58 (0.44,0.78)**	**0.52 (0.39,0.70)**	**0.63 (0.47,0.85)**	**0.71 (0.53,0.96)**
p for trend	**<0.001**	**<0.001**	**0.002**	**0.02**

Model 1: Adjusted for Age, Gender. Model 2: Further adjustment for Race/ethnicity, Marital status, PIR, BMI. Model 3: Further adjustment for Diabetes, Hypertension, Smoking status, Alcohol usage, Ldl-cholesterol, Energy take. The bold values mean statistical significance.

### 3.4 Relationship between dietary live microbes and OA

Univariate and multivariate logistic analysis of dietary live microbe content and the odds of OA were also carried out. As shown in Table 4, the findings from the univariate logistic regression analysis demonstrate a statistically significant correlation between the odds of OA and the intake of live microbes as part of the dietary regimen. Specifically, individuals in the medium and high dietary live microbe groups exhibited a significantly higher odds of OA compared to those in the low dietary live microbe group [Medium: OR 1.20, 95% CI (1.01, 1.43), High: OR 1.29 95% CI (1.04, 1.60)]. Furthermore, the odds of OA increased with a higher dietary intake of live microbes. However, upon controlling for the pertinent variables in a multivariate logistic analysis, it was determined that the difference in the odds of OA between the low dietary live microbe group and the mid and high dietary live microbe groups did not exhibit statistical significance. In all three models, there was no observed increase in the odds of OA with an increase in microbe intake.

**Table 4:** Association between levels of Dietary Live Microbe and OA.

	Crude model	Model 1	Model 2	Model 3
Low Dietary Live Microbe Group OR (95% CI)	1.00	1.00	1.00	1.00
Medium Dietary Live Microbe Group OR (95% CI)	**1.20 (1.01,1.43)**	0.94 (0.77,1.15)	0.94 (0.76,1.15)	0.95 (0.77,1.17)
High Dietary Live Microbe Group OR (95% CI)	**1.29 (1.04,1.60)**	1.08 (0.86,1.36)	1.04 (0.81,1.33)	1.10 (0.86,1.40)
p for trend	**0.02**	0.51	0.74	0.43

Model 1: Adjusted for Age, Gender. Model 2: Further adjustment for Race/ethnicity, Marital status, PIR, BMI. Model 3: Further adjustment for Diabetes, Hypertension, Smoking status, Alcohol usage, Ldl-cholesterol, Energy take. The bold values mean statistical significance.

### 3.5 Subgroup analysis

**Figure 2:**
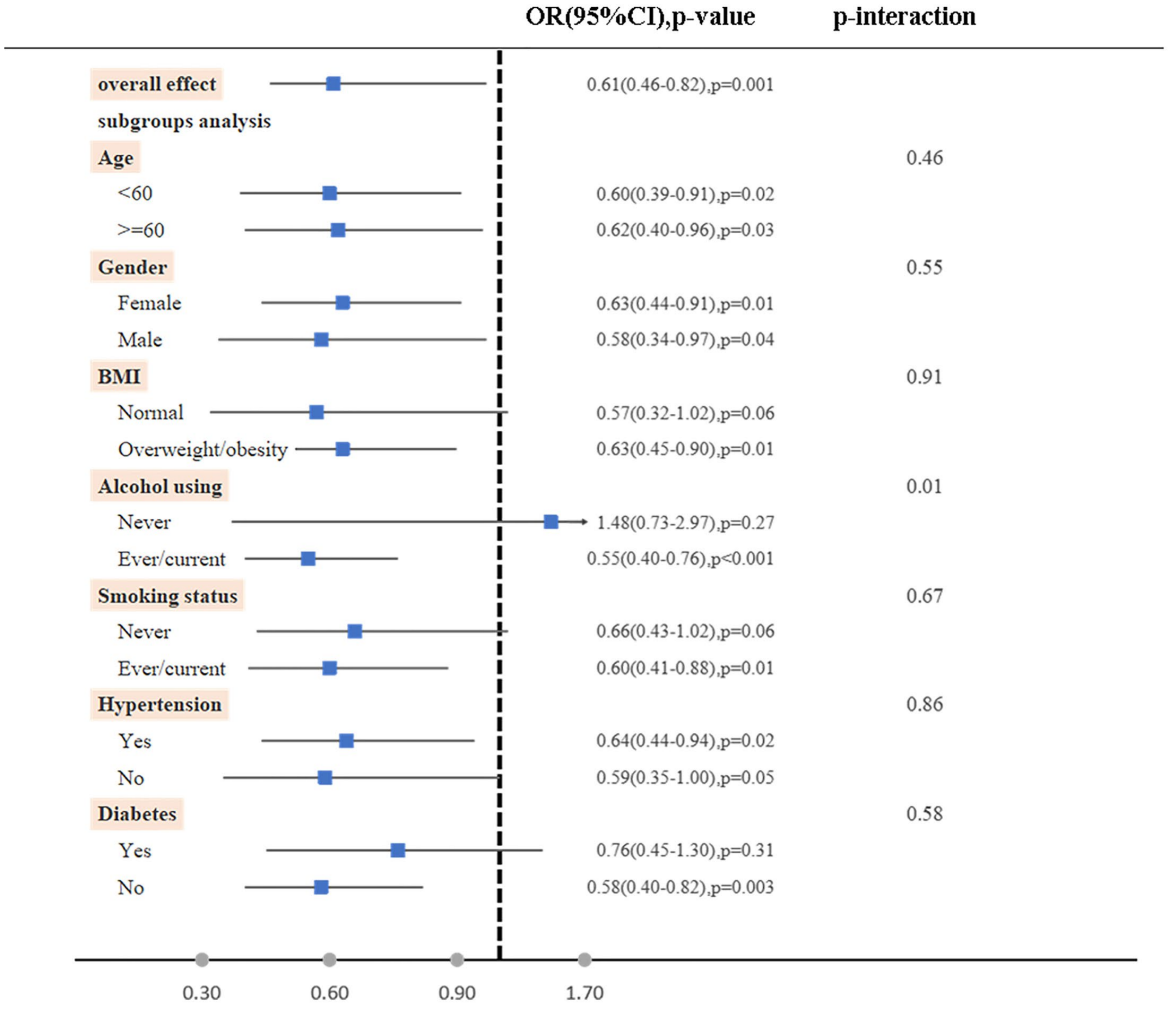
Subgroup analyses on the association between high vs. low dietary live microbe group with RA. The model was adjusted for age, gender, race/ethnicity, marital status, PIR, BMI, diabetes, hypertension, smoking status, alcohol using, Ldl-cholesterol, energy take.

Furthermore, it was found that when examining subgroups based on age, gender, BMI, alcohol using, smoking status, hypertension, and diabetes, the high dietary live microbe group exhibited a significantly lower odds of RA compared to the low dietary live microbe group. Notably, no significant interaction was observed between the dietary live microbes and these stratified variables, except for alcohol consumption (Figure 2).

### 3.6 Relationship between high dietary live microbes and odds of RA

**Figure 3:**
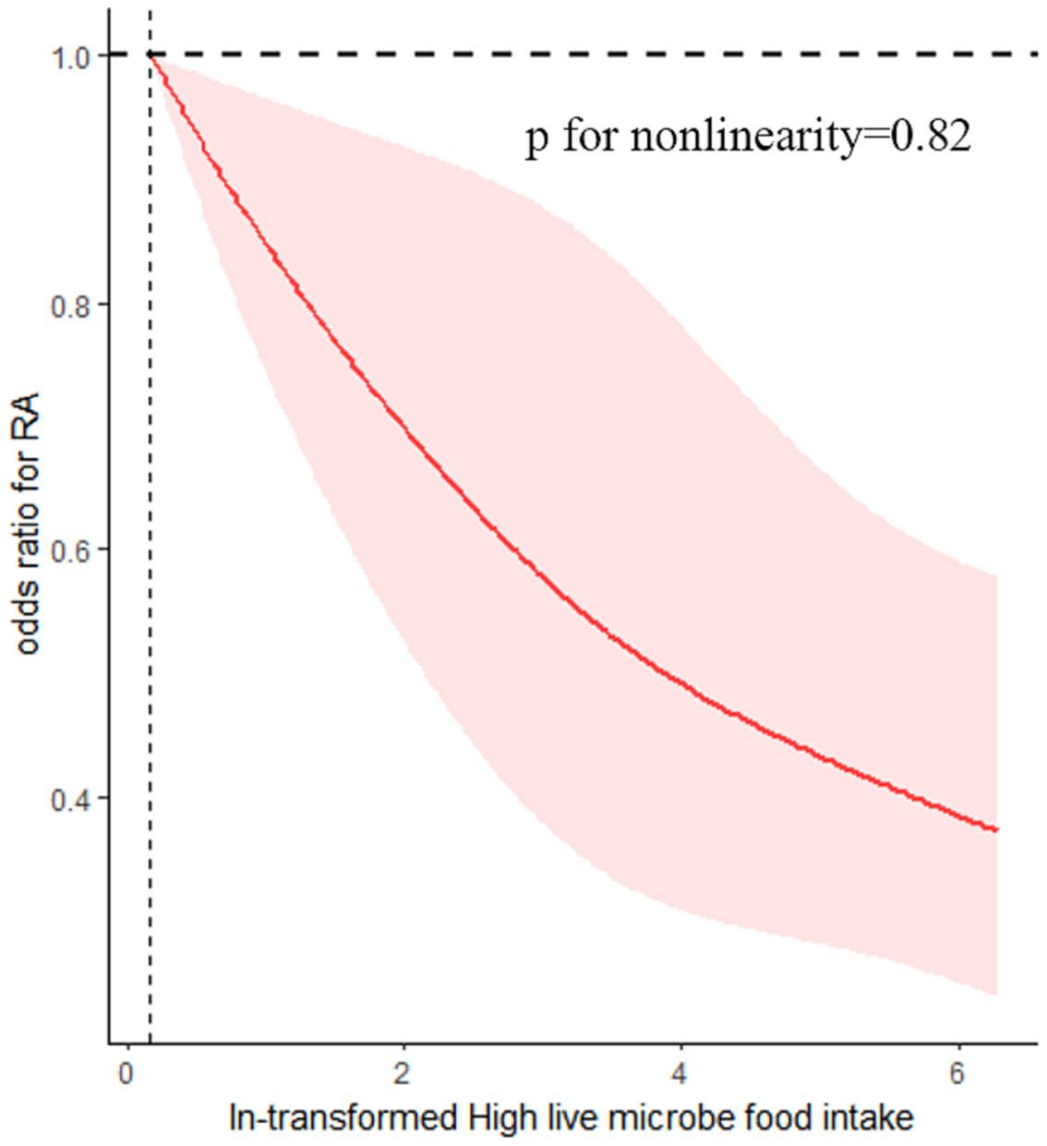
Association between dietary live microbes and odds of RA. The association was adjusted for age, gender, race/ethnicity, marital status, PIR, BMI, diabetes, hypertension, smoking status, alcohol using, Ldl-cholesterol, energy take.

The findings of this study indicate a negative dose–response relationship between the quantity of dietary live microbes and the occurrence of RA within the high dietary live microbe group (p for nonlinearity = 0.82). Specifically, an increase in the number of live microbes in food was associated with a decrease in the odds of developing RA (Figure 3).

## 4 Discussion

The intestinal tract can be considered an open ecosystem, where the consumption of microbes through dietary intake can influence the composition and behavior of the existing microbial population within the intestinal tract (24, 25). Several studies have posited that while the consumption of live food microorganisms may be eradicated by gastric acid, the portion that successfully reaches the intestinal tract might still exert a significant influence on the microbial ecosystem within the intestines. In the presence of low pH and elevated levels of the enzyme pepsin, many viable microorganisms undergo inactivation and subsequent death within the stomach and duodenum. However, when entering the small intestine and colon, these microbes began to resuscitate and grow due to the increase of PH and the infiltration of bile, trypsin and lipase. Therefore, although the inherent intestinal flora has a certain resistance to the colonization of foodborne microbes, the short-term fusion of foodborne microbes and intestinal intrinsic microflora cannot be ignored (26).

Based on a comprehensive examination of eight NHANES data cycles, our findings indicate that individuals who consume a diet rich in live microbes exhibit a reduced susceptibility to RA. However, our research did not reveal a statistically significant association between the quantity of live microbes in one’s diet and the odds of OA. The consumption of live microbes in food was found to be associated with a reduction in the odds of RA. The primary distinction between the high dietary live microbe group and the medium and low dietary live microbe group lies in the differential consumption of fermented food and probiotic supplements, with the former exhibiting a higher intake of these substances. Fermented food and beverages play a significant role in the human diet, constituting approximately one-third of dietary intake. Within these fermented products, microorganisms are a crucial component of the foodborne microbiota, with an estimated range of 10^8^–10^12^ CFU per day (26, 27). While fresh fruits and vegetables typically exhibit microbial biomass levels below 10^6^ CFU/g (28). A systematic review and meta-analysis of randomized control trials suggested that the intake of probiotic supplements has a positive impact on the reduction of IL-6 levels in individuals with RA, hence potentially regulating the progression of RA. However, additional clinical validation is required to substantiate these findings (29). In a similar vein, some studies have found that within the collagen-induced arthritis (CIA) model of Wistar rats, the administration of *Lactobacillus casei* has the potential to modulate the expression of COX-2, thereby mitigating the intensity of RA through the inhibition of the release of proinflammatory cytokines IL-6, IL-10, and TNF-α (30). Hence, there is a basis to posit that fermented foods and probiotic supplements could potentially exert a significant influence on the postponement and mitigation of the development of RA.

Probiotics mainly include Lactobacillus and Bifidobacterium, which reduce the pH of the intestinal tract and inhibit the growth of pathogenic bacteria by producing lactic acid and acetic acid (31). Some probiotics, such as *Lactobacillus casei* and *Lactobacillus acidophilus*, have anti-inflammatory, antibacterial, and antioxidant properties. These microbe symbiosis and colonization in the intestinal tract, maintain host nutritional support by increasing the integrity of the gastrointestinal tract, and reducing the severity of inflammatory diseases such as RA (14). Prior research has indicated that plant fermented food has anti-inflammatory and immunomodulatory properties, mainly due to the prebiotic effect of bioactive polyphenols produced by fermentation itself and the probiotic regulation of intestinal microflora (32–34). In addition, the metabolites of vegetables before and after lactic acid bacteria fermentation were analyzed by gas chromatography/time-of-flight mass spectrometry (GC/TOF-MS). It was found that the newly synthesized or depolymerized compounds with anti-inflammatory or antioxidant activities were significantly increased after fermentation, such as lactate, 3-phennyllactate, indole-3-lactate, β-hydroxybutyrate, γ-aminobutyrate, and glycerol and so on (35). In the collagen-induced arthritis model of Wistar rats, it was also proved that different kinds of Lactobacillus may regulate the balance of intestinal flora and their metabolites and regulate Th1/Th17-related immune responses to alleviate arthritis by inhibiting the release of proinflammatory cytokines or anti-CII antibody (36). Therefore, there is a rational basis to posit that a diet rich in live microbes could potentially mitigate the the odds of developing RA through anti-inflammatory and antioxidant effects.

The potential mitigation of RA severity by the consumption of a diet rich in living microorganisms is also supported by evidence suggesting that this dietary approach can lead to elevated levels of short-chain fatty acids (SCFA). Probiotics can enhance host immunity by increasing the level of SCFA, such as by inhibiting the pro-inflammatory NF-κ pathway and regulating Treg cells (37, 38). Butyrate is a kind of SCFA and has a positive effect on the treatment of RA. Some studies have suggested that butyrate may inhibit the expression of HDAC and the release of proinflammatory cytokines, then promote Treg polarization and suppress conventional T cells (Tconvs) and osteoclast differentiation, participate in the regulation of immune balance, and inhibit the severity of arthritis in RA (39, 40). Previous research has indicated that butyrate increases the level of AhR ligands (serotonin-derived metabolite 5-Hydroxyindole-3-acetic acid (5-HIAA)) through Regulatory B (Breg) cells dependence and reduces the severity of arthritis (41, 42). Hence, this factor may potentially serve as a potential explanation for the observed beneficial effects of a diet rich in live microbes on RA.

The findings of our study indicate that there is no statistically significant correlation between the consumption of dietary live microbes and the development of OA. At present, whether there is a causal effect between intestinal microbiome and OA is still controversial. While certain studies suggest that probiotic treatment may have the potential to attenuate cartilage destruction and enhance the subchondral bone structure in a preclinical model of osteoarthritis in mice undergoing destabilization of the medical meniscus (DMM), the precise underlying mechanism of its action remains uncertain (43). There is presently a sole clinical randomized controlled trial encompassing a total of 215 patients with *L. casei* Shirota and another 218 with placebo, which posits that probiotics might enhance the management of knee osteoarthritis by diminishing the levels of high-sensitivity C-reactive protein (hs-CRP) (44). Several studies suggest that probiotics may play a role in alleviating chronic pain associated with OA (45). Nevertheless, there is currently a dearth of scholarly evidence and large sample clinical studies documenting the precise correlation between probiotics and the susceptibility to OA.

The present research indicates that the consumption of a diet rich in live microbes may have a positive impact on mitigating the odds of RA. The human microbiome’s ability to be shaped, its incorporation into the immune system, and its sensitivity to dietary changes make it a compelling target for therapeutic intervention (4, 46). Ingesting live microorganisms through diet is less hazardous and has fewer adverse effects compared to pharmacological therapy (14). However, it is important to note that those with compromised immune systems and severe illnesses should exercise caution when considering the use of probiotics. Given the limited number of randomized controlled trials on probiotics and the lack of knowledge regarding the precise composition of bacteria in the food or items consumed by patients, it is advisable for these individuals to exercise caution when considering the supplementation of live dietary microbes (31).

The present study possesses certain limitations. First of all, it is important to note that this investigation adopts a cross-sectional design, which may restrict its ability to establish a definitive causal association between the consumption of live microbes in one’s diet and the development of arthritis. Furthermore, the classification methodology employed in this study to determine the content of live microbes in the diet has been developed by esteemed experts from various disciplines through collaborative discussions and extensive literature reviews. This approach offers notable advantages in terms of efficiency and applicability. However, it is important to acknowledge that, in comparison to directly measuring dietary microbial content, there may be a certain degree of error associated with this classification method. Lastly, it is worth noting that the research sample for this study is derived from the population of the United States, and therefore, it is necessary to validate the findings in other countries before drawing definitive conclusions.

## 5 Conclusion

This article conducted an exploratory analysis of the relationship between dietary live microbe intake and arthritis by analyzing the data of NHANES from 2003–2018. The results revealed a significant difference in dietary live microbe intake between healthy and RA patients. However, no significant association was found between the occurrence of OA and the quantity of dietary live microbes. Higher dietary intake was correlated with a decreased odds of RA, the consumption of a diet rich in live microbes may have a positive impact on mitigating the odds of RA.

## Data availability statement

The raw data supporting the conclusions of this article will be made available by the authors, without undue reservation.

## Ethics statement

The studies involving human participants were reviewed and approved by the National Center for Health Statistics (NCHS) Ethical Review Board. All participants provided written informed consent. The studies were conducted in accordance with the local legislation and institutional requirements.

## Author contributions

YL: Conceptualization, Formal analysis, Investigation, Methodology, Software, Writing – original draft. YX: Conceptualization, Data curation, Investigation, Writing – review & editing. XK: Project administration, Writing – review & editing.
