# Supplementary material for: Exploratory analysis on the relationship between dietary live microbe intake and arthritis: a national population based cross-sectional study
Source: Front Nutr. 2024 Jan 4;10:1328238. doi: 10.3389/fnut.2023.1328238 (PMC10794527; doi:10.3389/fnut.2023.1328238)
Supplement: Supplementary file 1 [file Table_1.DOCX]

Table S1. Table S1. Characteristics of final participants and excluded individuals with missing covariates (smoking staus, alcohol using, PIR, BMI, hypertension, diabetes, LDL-cholesterol)

|  | final participants  (n=12844) | participants with missing data  (n=21222) | p-value |
| --- | --- | --- | --- |
| **Age（%）** |  |  | **0.01** |
| <60 | 8822(74.95) | 14642(76.84) |  |
| >=60 | 4022(25.05) | 6580(23.16) |  |
| **Gender (%)** |  |  | 0.28 |
| Female | 6348(50.14) | 10612(50.95) |  |
| Male | 6496(49.86) | 10610(49.05) |  |
| **Race/ethnicity (%)** |  |  | **< 0.001** |
| Mexican American | 2086(8.06) | 3488(9.14) |  |
| Non-Hispanic Black | 2559(10.32) | 4667(11.77) |  |
| Non-Hispanic White | 5867(69.88) | 8847(65.74) |  |
| Other | 2332(11.74) | 4220(13.35) |  |
| **Marital status (%)** |  |  | 0.31 |
| Married/cohabiting | 7780(62.67) | 12582(61.76) |  |
| Widowed/divorced/separated/never married | 5064(37.33) | 8623(38.24) |  |
| **PIR (%)** |  |  | 0.23 |
| ≤1.35% | 3927(21.66) | 6063(22.98) |  |
| 1.35%-3.5% | 4789(34.75) | 6706(34.17) |  |
| ≥3.5% | 4128(43.58) | 5765(42.86) |  |
| **BMI (%)** |  |  | 0.1 |
| Normal | 3913(32.31) | 6220(31.08) |  |
| Overweight/Obesity | 8931(67.69) | 14637(68.92) |  |
| **Alcohol using (%)** |  |  | 0.06 |
| Ever/Current | 11167(89.53) | 15734(88.64) |  |
| Never | 1677(10.47) | 2694(11.36) |  |
| **Smoking status (%)** |  |  | 0.06 |
| Now/Former | 5755(45.56) | 9363(44.56) |  |
| Never | 7089(54.44) | 11842(55.44) |  |
| **Diabetes (%)** |  |  | **< 0.001** |
| No | 10494(85.86) | 17820(88.56) |  |
| Yes | 2350(14.14) | 3402(11.44) |  |
| **Hypertension (%)** |  |  | 0.24 |
| No | 7692(63.41) | 12597(64.37) |  |
| Yes | 5152(36.59) | 8620(35.63) |  |
| **Energy take(kcal)** | 2176.63±11.79 | 2164.78± 9.81 | 0.45 |
| **Ldl cholesterol (mg/dl)** | 114.59±0.46 | 113.33±1.08 | 0.25 |
| **Dietary live microbe group (%)** |  |  | 0.05 |
| Low | 4549(31.82) | 7687(33.03) |  |
| Medium | 5386(40.25) | 8926(40.90) |  |
| High | 2909(27.94) | 4609(26.07) |  |
| **Arthritis type (%)** |  |  | 0.27 |
| RA | 786(4.76) | 1286(4.44) |  |
| OA | 1577(13.29) | 2484(12.61) |  |
| HC | 10481(81.95) | 17452(82.95) |  |
